# Religiously Conditioned Health Behaviors within Selected Religious Traditions

**DOI:** 10.3390/ijerph19010454

**Published:** 2022-01-01

**Authors:** Anna Majda, Iwona Bodys-Cupak, Alicja Kamińska, Marcin Suder, Zofia Gródek-Szostak

**Affiliations:** 1 Laboratory of Theory and Fundamentals of Nursing, Faculty of Health Sciences, Institute of Nursing and Midwifery, Jagiellonian University Medical College, ul. Michałowskiego 12, 31-126 Krakow, Poland; anna.majda@uj.edu.pl (A.M.); alicja.kaminska@uj.edu.pl (A.K.); 2 Department of Applications of Mathematics in Economics, Faculty of Management, AGH University of Science and Technology, 30-067 Krakow, Poland; msuder@agh.edu.pl; 3 Department of Economics and Enterprise Organization, Cracow University of Economics, 31-510 Krakow, Poland; grodekz@uek.krakow.pl

**Keywords:** health behaviors, religiously, religious traditions

## Abstract

Background: Health is a value of paramount importance for human existence. It has a special place in every religious system, both on the doctrinal and practical levels. Most religions view health as a priority value to be cared for, and require followers of individual religious groups to take care of their physical and mental health, and to maintain a balance between body and spirit. The aim of the study was to verify whether the level of religious commitment significantly influences health behaviors and whether in selected religions the faithful have a different approach to health behaviors. Methods: This cross-sectional study was conducted on a group of 296 people—118 Seventh-day Adventists (SDA), 134 Catholics, and 14 Jews living in southern Poland, and 31 Muslims living in southern and north-eastern Poland. The following research tools were used as part of the diagnostic survey method: a questionnaire survey designed by us; the International Physical Activity Questionnaire (IPAQ); the Inventory of Health Behaviours (IHB); the Perceived Stress Scale (PSS 10) and anthropometric measurements, physical examination, laboratory tests. Results: Only 75% of Catholics who participated in the study declared a high level of religious commitment. On the other hand, all (100%) of SDA, Jews, and Muslim respondents declared their religious commitment at a high level. SDA were characterized by the most desirable health behaviors on the IHB (high and average levels), especially eating habits. They did not consume alcohol, did not smoke cigarettes. The physical activity of most ADS was high and moderate. Most of the SDA subjects were characterized by an average and low level of stress intensity. The most common correct scores for homocysteine, triglycerides, and CRP were SDA, cholesterol by Jews, glucose by Muslims, and HDL by Catholics. The most common negative results were: homocysteine, glucose and triglycerides among Jews, cholesterol and CRP among Catholics, HDL among Muslims. Optimal blood pressure was most common among Catholics, and hypertension was most often diagnosed among Jews. Most often, Muslims had the correct body weight, and at the same time it was the group of respondents most often diagnosed with obesity. In contrast, underweight was observed only among Catholics. The SDA subjects were most often overweight. Conclusions: The results suggest that public health professionals and nurses should develop culturally specific educational interventions, especially among Catholics.

## 1. Introduction

Religiousness and/or spirituality when it comes to health psychology is perceived as one of the elements of human development and refers to the needs of people when healthy and ill. It is also treated as the fourth dimension of health, next to the biological, mental, and social dimensions. The right to be healthy is equated with the right to live, regardless of origin, race, or religion.

In common awareness, religion and health are linked by a strong emotional relationship, associated with a sense of trust, entrusting God’s protection, with the simultaneous experience of helplessness in a situation of threat to health or life. Health is a value of imperative importance for human existence. It occupies a special place in every religious system, both on the doctrinal and practical level. Most religions perceive health as a gift from God and the superior value that should be taken care of, obliging the followers of particular religious groups to take care of their physical and mental health and maintain a balance between the body and spirit [1,2]. Religion serves various functions in terms of health: explicatory and meaning-creating function (it explains the phenomena of health and illness, death); normative and controlling function (it sanctions individual and social behavior towards health, illness, and death, sets the boundaries of medical experiments); caring and charity function (voluntary activities for the ill) and therapeutic, healing function. Common observations show that religious people care about health and life as a gift from God, following religious pro-health principles such as fasting, prohibition of stimulants, enduing ailments with optimism, and willpower supported by prayer activate the body to fight the illness. Religious beliefs can influence particular behaviors related to health practices. Patients may refuse to transfuse blood and blood-based products, use birth control, have an abortion due to religious beliefs, and health care workers may refuse to perform certain activities due to the conscientious objection [1,3,4,5,6].

People who practice religion differ in some consequences from those who do not do it. [7]. Religion supporting difficult rituals (for example, participation in church services, prayer, meditation, and fasting) and inhibitory beliefs (for example, belief in an omniscient, moralizing god who evaluates human behavior) strengthens control and improves people’s ability to self-regulate [8].

Many researchers assume that the impact of religiousness on health may be related to pro-health behaviors such as proper diet, physical activity, quitting smoking, or better compliance with medical recommendations. Additionally, regular observance of the holy days promotes rest, re-mobilization of physical and mental strength, and the organization of life goals and intentions [9]. However, the above factors do not fully explain the observed dependencies. It turns out that the positive impact of religiousness on health is also achieved through individual and community religious practices, such as meditation, prayer, rites of reconciliation, penance, and forgiveness—in other words, various acts of worship that strengthen positive feelings, such as hope, love, satisfaction, forgiveness and reduce negative emotions such as hostility. Ritual meetings within a specific community can also serve the functions of psychotherapy and sociotherapy, being a source of support and a sense of belonging to a larger social unit. The mechanism of the influence of religiousness through feelings and emotions can also be based on physiological foundations because it is known that positive emotions reduce the activity of the sympathetic part of the autonomic nervous system and the hypothalamic-pituitary-adrenal axis (thus reducing the secretion of stress hormones such as noradrenaline and cortisol), which has both psychological (reducing anxiety) and physiological effects (lowering blood pressure, reducing heart rate and oxygen consumption) and that way it may contribute to better overall health [9,10]. The model of the influence of psychosocial factors on physical health through neuroendocrine and immunological mechanisms, which causes changes at the molecular and cellular level, is already well established in health psychology [11]. These are probably not the only mechanisms, and there are undoubtedly many other psychological, behavioral, and biological factors that play a role in this effect and require further research.

There are not many publications in Polish scientific literature on the results of research on the relationship between religion and health, although the interest in this matter among public health and preventive medicine professionals is increasing [4,5,12]. Some research results confirm the positive influence of religion on human health. In the groups of more religious people, we can observe, inter alia, lower levels of anxiety, higher resistance to stress, reduced risk of cardiovascular diseases, and lower mortality rates in the course of chronic diseases [13,14].

A review of foreign literature regarding religious affiliation and disease did not show clear relationships between these variables. Some epidemiological studies conducted worldwide suggest a beneficial influence of religiousness when it comes to reducing the incidence of cardiovascular diseases, cancer, and prolonging life [15]. For example, studies among Seventh-day Adventists have shown the influence of religious practice on the reduction of the mortality rate [16,17,18,19,20]. Williams [21] has shown in studies that there is a relationship between religious activities and mental health. Another study [22] investigated the relationship between religion, mental health, and religiousness. Rippentrop [23] examined the relationship between religion, mentality, physical and mental health in sick people suffering from long-term pain. The multiple regression test results showed that there is a significant relationship between religiousness as a coping strategy and treatment and reduction of physical pain. Religious people are also less prone to use addictive substances, which was shown by the research carried out in the 1970s [24], and subsequent researches carried out in the 1990s confirmed these dependencies [25]. More religious patients also cope better with diseases, suffering, and disabilities [26,27]. However, there are also studies that show no relationship between religiousness and health [28,29].

The Catechism of the Catholic Church (CCC) emphasizes the value of physical health and calls to care for the health of citizens (and thus for public health). Perceiving health as a fundamental value enabling the realization of other good things, also states that it is not an absolute value and warns against the cult of the body [30,31]. The Catholic Church emphasizes the need to care for health by criticizing the use of drugs and abuse of alcohol. When examining the teaching of the Catholic Church in terms of the value of health and illness, it should be noted that, contrary to the modern belief of the medical world on the harmfulness of nicotine, the CC states that moderate tobacco use is not an inappropriate activity (harmful and therefore sinful, is only abusing it—the same applies to alcohol) [32]. People are obliged to care for their health properly. In the event of being sick, it is absolutely obligatory for a person to regain health. Therefore it becomes absolutely obligatory to follow the recommendations regarding treatment, care, rehabilitation, and diagnostics [33]. Judaism perceives man as a unity of body and soul and imposes on a man the obligation to care for his body and thus for health. Birth, taking care of a newborn, and circumcision are subject to numerous hygiene regulations. There are associations whose members care for the sick. It is religiously obligatory to wash your hands after waking up, before eating, after leaving the toilet, or after cutting nails. Maintaining an appropriate rhythm of life is of decisive importance for mental health, in which the celebration of the Sabbath plays a crucial role [33,34].

The greatest Jewish doctor, philosopher, and theologian—Moses ben Maimon, who in his works on hygiene, dietetics, and psychosomatics, strongly emphasized the value of prevention (especially physical exercise and proper diet) [33,34]. In Islam, the condition for the health of the body and soul is faith in God and faithful submission to His will. Many provisions of Islamic law relate to health issues, such as the obligation to fast, regular prayer (“to calm hearts”), or pilgrimage, which is to relieve everyday tensions and troubles and give a sense of community. In Islam, preventive measures include the prohibition of drinking alcohol, smoking cigarettes, using drugs, eating pork. It is recommended to play sports, eat properly and rest. The “prophetic medicine” was created based on preserved traditions of healing methods and statements of the Prophet Muhammad. According to the followers of the Seventh-day Adventist Church, one should provide the body with an adequate level of exercise and rest, eat the healthiest diet possible, and refrain from foods identified as unclean in the Scriptures. Since alcoholic beverages, tobacco and the misuse of medications as well as drugs are harmful to our body, one should abstain from them. Instead, everything that leads the mind and body to obedience to Christ, who wants life to be wholesome, happy, and good, should be included [35]. Researchers emphasize that in creating a spiritual meaning in the modern world, Adventism is an external reference to social change, that the understanding of religion should be expanded through the theory of new media [36,37].

This article analyzes the followers of the following religious groups: Catholics (CA), Seventh-day Adventists (SDA), Muslims (M), Jews (J). According to the Central Statistical Office, the Catholic Church (CC) is the largest religious community in Poland, with approximately 32 million baptized in 2018, but with only 38% participating in masses. About 10–12 thousand Muslims, 8–12 thousand Jews, and about 10 thousand Seventh-day Adventists (SDA) live in Poland [https://www.biing.com/, accessed on: 14 March 2021].

## 2. Materials and Methods

### 2.1. The Aim of the Study

The article has two main goals. The first was to verify whether the level of religious commitment significantly influences health behaviors. The second was to diagnose whether, in selected religions, the followers have a different approach to health behaviors.

### 2.2. Study Organization and Course

In 2014–2017 cross-sectional research was conducted among 296 followers of selected religions as part of the statutory project K/ZDS/004688. The selection of the study group was purposeful. Some of the research results from this project have been published [36]. The J and M were reached by announcements about the conducted research. The dates were set by representatives of the Jewish Community, the Muslim Center, and the Muslim Religious Union based in Poland. The respondents were recruited from among men and women living in southern Poland and Podlasie. Oral and written announcements informing about the conducted research were reaching the CA and SDA, and their schedule was set by pastors and priests. CA and SDAs were recruited around the southern part of Poland. The respondents were guaranteed anonymity. The study participants were informed both orally and in writing about the assumptions of the study as well as its course, voluntary participation, and the possibility of withdrawing from participating in the study at any stage without suffering any consequences. The respondents provided their consent for participation in the study by completing and signing the “Respondent consent form” and the “Processing of personal data form” before starting the project. Practitioners of particular religions were identified by making a statement.

The criteria for inclusion in the study was: age over 18 and being a declared Catholic, SDA, Judaist, or Muslim. Exclusion criteria were, declaring atheism or religion other than Catholicism, SDA, Judaism, Islam, being pregnant, breastfeeding, autoimmune diseases, cancer, surgeries within the last three weeks. In the body where there are deficiencies of the components necessary for the metabolism of homocysteine (folic acid, vitamin B6 and B12), this amino acid enters the bloodstream, causing hyperhomocysteinemia. As a consequence, the walls of blood vessels are damaged, and atherosclerosis develops and intensifies. Also, CRP, produced in response to inflammation developing in atherosclerosis, is recommended as an indicator of the risk of cardiovascular diseases—CVD. Therefore, among others the concentration of these two biochemical parameters in the blood was examined as new risk factors for CVD among selected religious denominations. Since increased CRP in the blood is also characteristic of: infections, autoimmune inflammatory diseases, neoplastic diseases, injuries and invasive procedures, it was decided to exclude people with these disorders from the study. Folic acid deficiency, which may occur in pregnant women and nursing mothers and cause hyperhomocysteinemia, was another reason for exclusion from the study [38,39].

### 2.3. The Study Group

The study of cross-sectional nature was conducted on a group of 296 people—118 Seventh-day Adventists (SDA), 134 Catholics (CA), 14 Jews (J) living in southern Poland, and 31 Muslims (M) living in southern and north-eastern Poland (Table 1). Only 75% of Catholics who participated in the study declared a high level of religious commitment. On the other hand, all (100%) of SDA, Jews, and Muslim respondents declared their religious commitment at a high level.

### 2.4. Methods, Techniques and Research Tools

The research tools that used as part of the diagnostic survey method included: a questionnaire survey designed by us; the International Physical Activity Questionnaire (IPAQ) developed by M. Sjöström, B. Ainsworth, A. Bauman, F. Bull, C. Craig, and J. Sallis [40]; the Inventory of Health Behaviours (IHB) by Z. Juczyński [41]; the Perceived Stress Scale (PSS 10) by S. Cohen, T. Kamarck, and R. Mermelstein, adapted by Z. Juczyński, and N. Ogińska-Bulik [42] and anthropometric measurements, physical examination, laboratory tests. Details of the research methodology are included in the article “Comparison of Lifestyle of Catholics and Seventh-Day Adventists and the Relationship with Homocysteine as Risk Factor for Cardiovascular Diseases, A Cross-Sectional Study in Polish Males and Females” [43].

In order to identify whether there are differences between the studied groups in the scope of the analyzed indicators, the percentage distributions of frequency of the levels of the analyzed variables were presented. Verification of whether these distributions differ significantly was performed using the Chi-square test of independence—Chi2 (Pearson’s test). The significance level was equal to *p* < 0.05 [44].

The following specific objectives have been set in this study. Based on the available data, it was decided to:

1. verify whether the declared level of religious commitment among Catholics is a factor differentiating their health behaviors.
2. verify whether the overall health (determined by selected parameters) of Catholics differs depending on the level of religious commitment declared by them.
3. determine whether there is a differentiation among the religious groups in terms of health behaviors.
4. determine whether the indicated differences in health behaviors of people of particular faiths are reflected in their overall health (determined by selected parameters).

### 2.5. Ethical Considerations

The Academic Bioethics Committee (KBET/79/B/2014) issued the ethical approval. Participants were provided a document that included information on the purpose, benefits, potential risk, and voluntary withdrawal from the study. The research protocol was prepared prior to KBET’s decision. The study was designed, carried out, and the results prepared in accordance with the principles of (1) Good Scientific Practice, (2) the Act of 10 May 2018 on personal data protection, (3) the Declaration of Helsinki and in accordance with (4) Regulation of the European Parliament and Regulation (EU) 2016/679 of 27 April 2016. on the protection of individuals with regard to the processing of personal data and on the free movement of such data, and (5) the repeal of Directive 95/46/EC (general regulation about data protection). The participants of the study were provided with all necessary information about the study, they were informed, among others, about the purpose of the study, ensuring anonymity, voluntary participation in it, and the possibility of withdrawing from participation in it at every stage of its conduct, without giving any consequences and reasons for refusal.

## 3. Results

### 3.1. Health Behaviors and Overall Health of Catholics with High and Low Levels of Religious Commitment

**Figure 1:**
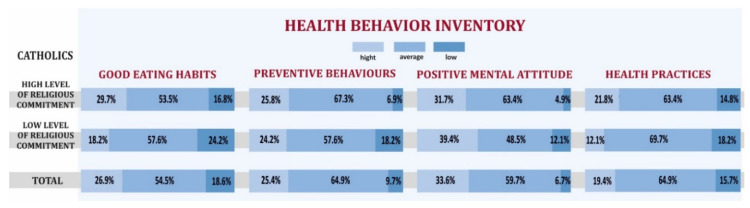
Cumulative frequencies of individual IHB indicators broken down into categories depending on the level of religious commitment of Catholics.

To take into account the assessment of religious commitment among Catholics, this religious group was divided into people with a declared high and low level of religious commitment, therefore in the first part of the study an attempt was made to compare the health behaviors of people from these two groups. As already mentioned, the analysis of health behaviors concerned several aspects described previously. Therefore, it was examined what percentage of the respondents defined their health behavior at a certain level of religious commitment for each of them. Figure 1 shows the distribution of behavior of Catholics for individual indicators in the IHB, divided into Catholics with high and low levels of religious commitment. Additionally, the cumulative distribution of indicators for all Catholics in total was provided. From the figure, we can read that when it comes to the good eating habits indicator, nearly 30% of highly religiously committed Catholics were classified as having a high level of behavior in this regard. For comparison, among Catholics with a low level of religious commitment, approximately 18% of respondents reached this level of behavior. In this category of behaviors, over 24% of Catholics with a low level of religious commitment fell into the group with a low rate of positive behavior. For comparison, among Catholics with a high level of religious commitment, this percentage was close to 17%. Therefore, we can conclude that religiously committed Catholics did much better in terms of good eating habits, than those who were not as religiously committed. Likewise, Catholics with a high level of religious commitment did better in terms of preventive behaviors (25.8% vs. 24.2%) and health practices (21.8% vs. 12.1%). However, Catholics with high levels of religious commitment scored lower on positive mental attitude (31.7%) than Catholics with low levels of religious commitment (39.4%). The above analysis shows that in the case of three of the four IHB indicators, the behavior of Catholics with a high level of religious commitment was at a higher level than in the group of Catholics with a low level of religiousness. The differences indicated in the distributions were not statistically significant (all *p*-values for the chi-square test are greater than 0.05).

**Figure 2:**
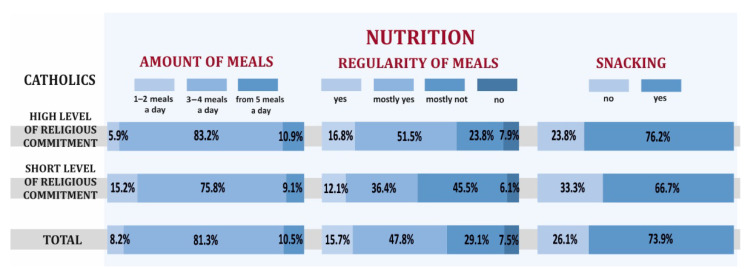
The frequency, regularity of meals, snacks between meals depending on the level of religious commitment of Catholics.

The Catholics with a high level of religious commitment who participated in the study consumed 3–4 meals a day (83.2% vs. 75.8%), paid attention to the regularity of meals (16.8% vs. 12.1%) more than Catholics with a low level of religious commitment, who in turn did not have snacks between meals as often (33.3% vs 23.8%). Also, in the case of eating behaviors, Catholics showed more healthy behaviors, but the presented differences were also not statistically significant (Figure 2).

**Figure 3:**
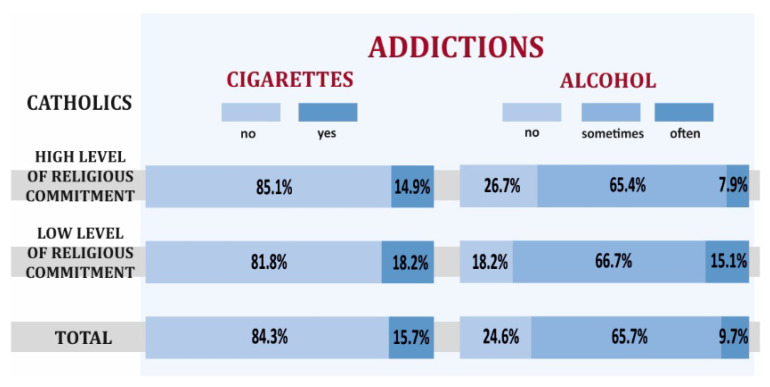
Smoking cigarettes, alcohol consumption depending on the level of religious commitment of Catholics.

The surveyed Catholics with high religious commitment more often did not smoke cigarettes (85.1% vs.81.8%) and did not drink alcohol (26.7%) compared to Catholics with a low level of religious commitment (18.2%). These results may also lead to the assumption that more religiously committed people used stimulants less frequently. The demonstrated differentiation in the use of stimulants such as alcohol and nicotine turned out to be statistically insignificant (Figure 3).

**Figure 4:**
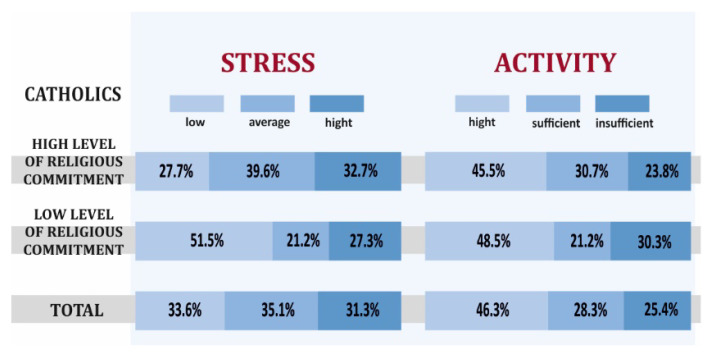
Stress level and physical activity depending on the level of religious commitment of Catholics.

Surveyed Catholics with a low level of religious commitment were less likely to show a high level of stress (27.3% vs. 32.7%), and more often, a high level of physical activity (48.5% vs. 45.5%) (Figure 4).

**Figure 5:**
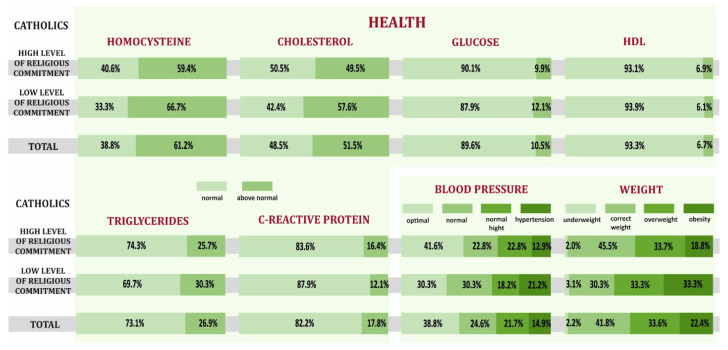
Biochemical values, blood pressure, body weight depending on the level of religious commitment of Catholics.

The overall health of Catholics through the prism of selected biochemical values in blood serum, blood pressure, and body weight. Catholics who were declaring a low level of religiousness had unhealthy levels of homocysteine (66.7% vs. 59.4%), cholesterol (57.6% vs. 49.5%), glucose (12.1% vs. 9.9%), triglycerides (30.3% vs. 25.7%), and only slightly better results in test of HDL and CRP. Catholics who were declaring a high level of religiousness more often had optimal blood pressure (41.6% vs. 30.3%) and normal body weight (45.5%) compared to Catholics with a low level of religiousness (30.3%). Summarizing the overall health analysis, we can state that Catholics with a high level of religious commitment had better parameters of indicators determining the overall health (Figure 5).

The comparative analysis of the health behaviors and overall health of two groups of Catholics (with a high level of religious commitment and a low level of religious commitment) shows the noticeable difference for these groups in the aspects taken into consideration. Generally, in the first group of Catholics, a greater percentage of people showed positive health behaviors. These positive health behaviors translated into appropriate indicators of health-related parameters. Admittedly, the observed differentiation was not statistically significant. This could be due to the fact that the group of people who underwent the tests was too small. Only Catholics with a low level of religious commitment experienced high levels of stress less often and engaged more often in high levels of physical activity.

### 3.2. Health Behaviors and Overall Health of Representatives of Selected Religious Groups with a High Level of Religious Commitment

**Figure 6:**
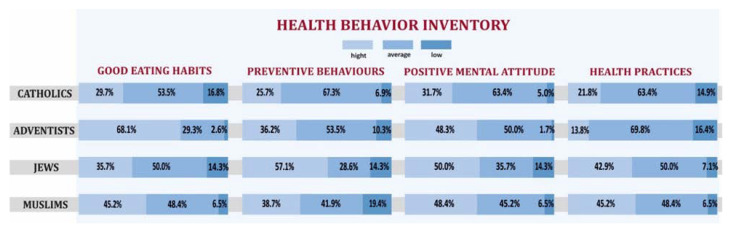
Cumulative frequencies of IHB indicators broken down by categories in individual religious groups declaring high level of religiousness.

As already mentioned, the second part of the study was related to the comparison of health behaviors of people of different religious groups. Only people who declared high commitment to religious practices were taken into account in the analysis. Figure 6 shows that SDA had by far the best eating habits in IHB. Among ADS, nearly 70% of respondents indicated positive behaviors in this regard. For comparison, among Muslims, such persons constituted slightly more than 45%, and among Catholics, there were less than 30%. When comparing behaviors in the field of prevention, Jews definitely did the best, with over 57% of them having highly positive behaviors in this regard. SDA and Muslims had a similar result, while Catholics had the worst result (25%). Also, in the case of a positive attitude, Catholics significantly deviated from other religions. Among Catholics, a high level of positive attitude was shown for nearly 32% of respondents, and for other religions, this indicator was close to 50%. In terms of health practices, the best behaviors were demonstrated by Muslims and Jews (high levels were shown for over 40% of respondents, however, the worst results in this regard were achieved by SDA (13.8% of high indications), and only slightly higher results were achieved by Catholics (21.8% of high indications). In order to verify whether the observed differences for the indicators of IHB in particular religious groups were statistically significant, an independence test was performed. It turned out that, the observed differentiation turned out to be statistically insignificant only in the case of a positive attitude. In the remaining cases, the differences were statistically significant (the obtained *p*-values for the test of independence were 0.000012 for good habits, 0.045 for preventive behaviors, and 0.0036 for health practices, respectively).

**Figure 7:**
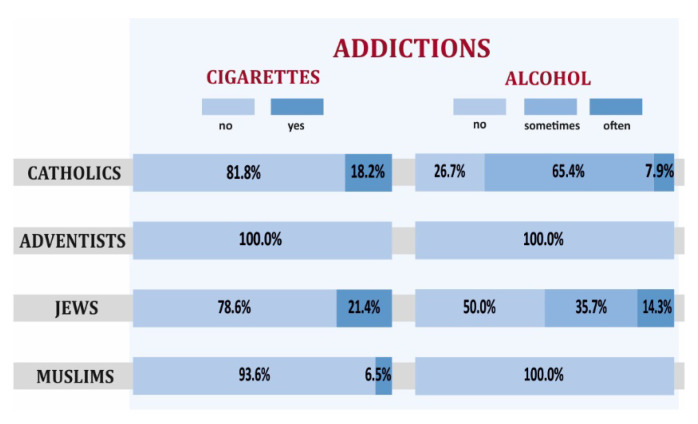
Smoking cigarettes, drinking alcohol in selected religious groups declaring high level of religiousness.

Risky health behaviors such as drinking alcohol and smoking cigarettes were most severe among Jews and Catholics. It is noteworthy that none of the respondents among SDA was smoking or drinking alcohol. Both in the case of smoking cigarettes and drinking alcohol, the differences noticeable in Figure 7 are statistically significant (for smoking cigarettes, the *p*-value of the test probability was 0.00001, while for drinking alcohol, it was 1.3 × 10^−10^).

**Figure 8:**
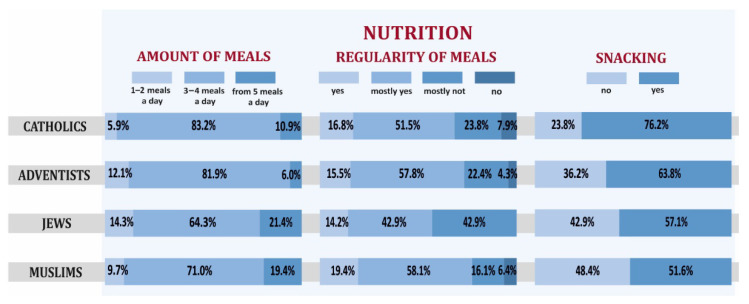
The frequency and regularity of meals, snacking between meals among particular religious groups declaring high level of religiousness.

Among eating habits, the regularity and frequency of eating meals as well as snacking between main meals were examined. As shown by the analyses, most often 3–4 meals a day were consumed by Catholics, 5 meals by Jews (Figure 8). The regularity of eating meals in the highest percentage of respondents was observed among Muslims while snacking between meals among SDA. It should be noted that the observed differences were not large and turned out to be statistically insignificant.

**Figure 9:**
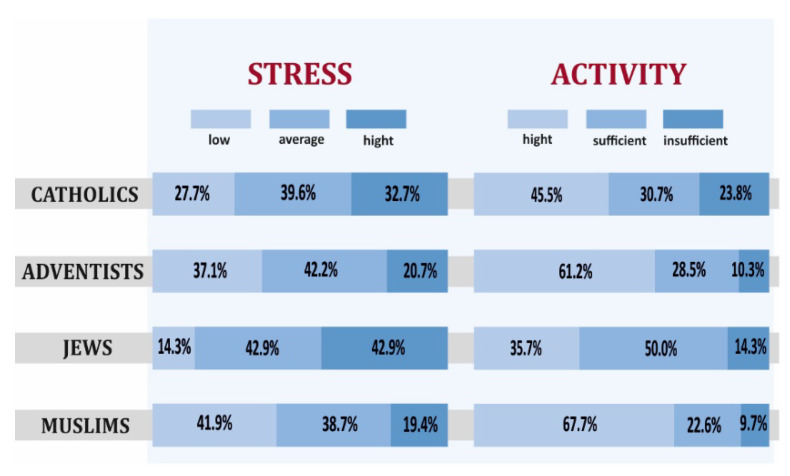
Level of stress and physical activity in individual religious groups declaring high level of religiousness.

The Jews surveyed had the highest level of stress on the PSS-10 scale, and Muslims—the lowest (Figure 9). The analysis of The International Physical Activity Questionnaire showed that the group with the most frequent high rate of physical activity were Muslims, and the group with the lowest rate—Catholics. The differentiation in terms of stress turned out to be statistically insignificant, and in the case of physical activity, it turned out to be significant because the obtained *p*-value was 0.0109, which means it was lower than the adopted level of 0.05.

**Figure 10:**
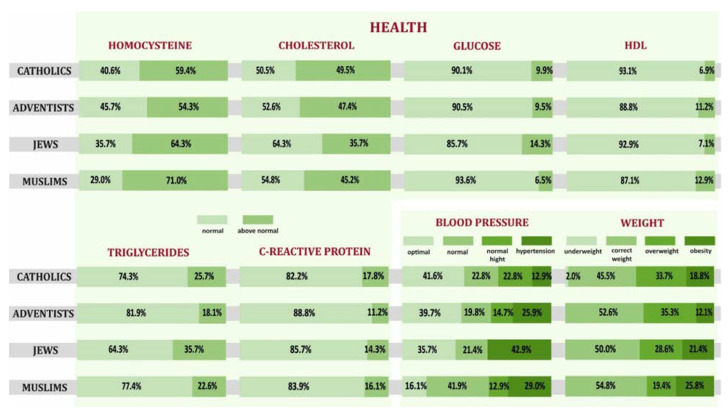
Biochemical values, blood pressure, body weight in individual religious groups declaring high level of religiousness.

The overall health of the participants of the study was assessed through the prism of selected biochemical values in blood serum, blood pressure, and body weight. The analysis of the data showed that the homocysteine, triglycerides, and CRP results within the norm were most often demonstrated by ADS, cholesterol—Jews, glucose—Muslims, and HDL—Catholics. The most often negative results were: homocysteine, glucose, and triglycerides among Jews, cholesterol, and CRP among Catholics, HDL among Muslims. Optimal blood pressure was most often demonstrated among Catholics, and hypertension was most often diagnosed among Jews. Muslims most often had the optimal bodyweight, but at the same time, it was the group of respondents most often diagnosed with obesity. On the other hand, instances of being underweight were observed only among Catholics. The SDAs who participated in the study were most often overweight (Figure 10).

## 4. Discussion

An important element shaping health risk is the inclusion of elements related to a healthy lifestyle in the professed religious values [45]. Attempts to assign the risk of developing the disease to specific religious denominations (communities) in the medical literature were made at the beginning of the 20th century, mainly in the United States [44,46,47,48]. These issues are still of interest and scientific elaboration, especially in European countries [49,50,51,52,53,54]. In the world literature one can find studies on the health behaviors of SDA, Muslims, and Jews, but there are no studies concerning Catholics.

Researchers want to find out which elements of a lifestyle based on religious commitment are important for health. They ask themselves how eating habits, addictions, physical activity, and exposure to stress affect the risk assessment of disease among followers of different religions. This phenomenon was first investigated in the 1980s also in Poland [12]. The role of religiousness when it comes to coping with various health and illness situations has begun again to be recognized in Polish literature in recent years [55]. Although there is still an insufficient amount of research on the subject due to the low number of SDAs, Muslims, Jews, and Catholics (although they constitute the largest percentage of religious followers in Poland) the results of the presented study are a perfect complement to them, despite the fact that it is impossible to start a discussion in the absence of research results of other Polish authors. Also, foreign authors did not consider these issues through the prism of, for example selected lifestyle elements or biochemical, anthropometric indicators, and blood pressure values, that affect the risk of cardiovascular diseases, as the authors of the article.

In the presented research, the results correspond with the results of other researchers. As in other epidemiological studies, SDAs [45] were characterized by the most desirable health behaviors on the IHB (high and average levels), especially when it comes to eating habits. They did not consume alcohol, did not smoke cigarettes. The physical activity of most SDAS was high and moderate. Most of the SDA respondents were characterized by an average and low level of stress intensity.

The respondent’s overall health was assessed through the prism of selected biochemical values in blood serum, blood pressure, and body weight. The study provided evidence that homocysteine, triglyceride, and CRP results within the normal range were obtained most often by SDA, cholesterol—Jews, glucose—Muslims, and HDL—Catholics. The most common negative results were: homocysteine, glucose, and triglycerides among Jews, cholesterol, and CRP among Catholics, HDL among Muslims. Optimal blood pressure was most common among Catholics, and hypertension was most often diagnosed among Jews. Most often, Muslims had the optimal bodyweight, but at the same time, it was the group of respondents most often diagnosed with obesity. On the other hand, being underweight was observed only among Catholics. The SDAs were most often overweight. No results from similar studies were found to be able to compare them.

The strength of this study was not only learning about the health behaviors declared by the respondents but also diagnosing their health through anthropometric measurements, blood pressure measurements, and blood sampling for selected biochemical indicators. We recognize that the main limitations of this study were that the study was located in one place and had a small sample size. The limitations typical of the methodology, controversy related to the cultural determinants of various religious traditions, and disruptive factors (such as age, gender, the influence of changing social approval), require that these results to be treated with caution. Further research can be extended to many places. We recommend repeating the study on a larger sample.

## 5. Conclusions

1. Only 75% of surveyed Catholics declared high religious commitment only, meanwhile for the surveyed SDA, Muslims, and Jews it was 100%.
2. Catholics with a high level of religious commitment declared more positive health behaviors and presented better overall health compared to Catholics with a low level of religiousness, but also higher levels of stress, worse mental attitude, and less physical activity.
3. Selected elements of lifestyle, overall health through the prism of the results of biochemical and anthropometric tests, blood pressure of Jews and Muslims differed from the recommendations of a healthy lifestyle. Still, they differed the most for Catholics and the least for SDAs.
4. The results suggest that public health professionals and nurses should develop culturally specific educational interventions for the prevention cardiovascular disease, especially among Catholics, concerning a healthy lifestyle, including a change in diet, physical activity, stress reduction, and elimination of addictions.

## Figures and Tables

**Table 1:** Sociodemographic data.

Variables		Muslims		Jews		Catholics		SDA	
		N-31	%	N-14	%	N-134	%	N-118	%
Level of religious commitment	High	31	100	14	100	101	75.4	118	100
	Low	0	0	0	0	33	24.6	0	0
Sex	Female	18	58.1	10	71.4	87.0	64.9	75.0	63.6
	Male	13	41.9	4	28.6	47.0	35.1	43.0	36.4
Place of residence	Village	4	12.9	0	0.0	29.0	21.6	39.0	33.1
	City	27	87.1	14	100.0	105.0	78.4	79.0	66.9
Education	Primary/Elementary	1	3.0	1	7.0	7.0	5.2	6.0	5.1
	Vocational	6	19.4	0	0,0	18.0	13.4	32.0	27.1
	Secondary	4	12.9	2	14.0	33.0	24.6	41.0	34.7
	Higher	20	64.5	11	78.6	79.0	56.8	39.0	33.1
Professional activity	Physical work	4	12.9	1	7.0	17.0	12.7	39.0	33.1
	Intellectual work	16	51.6	7	50.0	90.0	67.2	43.0	36.4
	Unemployed	11	35.5	6	42.6	27.0	20.1	36,0	30.5
Source of income	Professionally active	19	61.3	7	50.0	94.0	70.2	75.0	63.6
	Disablement pension	0	0.0	1	7.0	6.0	4.5	7.0	5.9
	Retirement pension	9	29.0	5	35.7	30.0	22.4	32.0	27.1
	Benefits	0	0.0	0	0.0	4.0	2.9	4.0	23.4
		Range	M (SD)	Range	M (SD)	Range	M (SD)	Range	M (SD)
Age		21–84	52.0 (17.5)	22–82	53.9 (23.1)	20–96	47.7 (16.0)	24–94	53 (15.8)

Key: N—number of subjects; M—mean; SD—standard deviation.

## Data Availability

Not applicable.
